# Evidence on the Human Health Effects of Low-Level Methylmercury Exposure

**DOI:** 10.1289/ehp.1104494

**Published:** 2012-01-24

**Authors:** Margaret R. Karagas, Anna L. Choi, Emily Oken, Milena Horvat, Rita Schoeny, Elizabeth Kamai, Whitney Cowell, Philippe Grandjean, Susan Korrick

**Affiliations:** 1 Section of Biostatistics and Epidemiology, Geisel School of Medicine at Dartmouth, Hanover, New Hampshire, USA; 2 Department of Environmental Health, Harvard School of Public Health, Boston, Massachusetts, USA; 3 Department of Population Medicine, Harvard Pilgrim Health Care Institute and Harvard Medical School, Boston, Massachusetts, USA; 4 Department of Environmental Sciences, Jožef Stefan Institute, Ljubljana, Slovenia; 5 U.S. Environmental Protection Agency, Washington, DC, USA; 6 Channing Laboratory, Department of Medicine, Brigham and Women’s Hospital, Harvard Medical School, Boston, Massachusetts, USA

**Keywords:** birth outcomes, cardio-vascular disease, epidemiology, health outcomes, low-level exposure, metals, methylmercury, neuro-logic outcomes

## Abstract

Background: Methylmercury (MeHg) is a known neuro-toxicant. Emerging evidence indicates it may have adverse effects on the neuro-logic and other body systems at common low levels of exposure. Impacts of MeHg exposure could vary by individual susceptibility or be confounded by bene-ficial nutrients in fish containing MeHg. Despite its global relevance, synthesis of the available literature on low-level MeHg exposure has been limited.

Objectives: We undertook a synthesis of the current knowledge on the human health effects of low-level MeHg exposure to provide a basis for future research efforts, risk assessment, and exposure remediation policies worldwide.

Data sources and extraction: We reviewed the published literature for original human epidemio-logic research articles that reported a direct biomarker of mercury exposure. To focus on high-quality studies and those specifically on low mercury exposure, we excluded case series, as well as studies of populations with unusually high fish consumption (e.g., the Seychelles), marine mammal consumption (e.g., the Faroe Islands, circumpolar, and other indigenous populations), or consumption of highly contaminated fish (e.g., gold-mining regions in the Amazon).

Data synthesis: Recent evidence raises the possibility of effects of low-level MeHg exposure on fetal growth among susceptible subgroups and on infant growth in the first 2 years of life. Low-level effects of MeHg on neuro-logic outcomes may differ by age, sex, and timing of exposure. No clear pattern has been observed for cardio-vascular disease (CVD) risk across populations or for specific CVD end points. For the few studies evaluating immunologic effects associated with MeHg, results have been inconsistent.

Conclusions: Studies targeted at identifying potential mechanisms of low-level MeHg effects and characterizing individual susceptibility, sexual dimorphism, and non-linearity in dose response would help guide future prevention, policy, and regulatory efforts surrounding MeHg exposure.

Methylmercury (MeHg) from natural or anthropogenic sources biomagnifies through the food chain and gives rise to human exposure primarily through consumption of higher trophic level fish and marine mammals [National Research Council (NRC) 2000]. MeHg crosses the placenta and readily passes through the blood–brain barrier, with even higher MeHg levels in fetal than in maternal circulation (Stern and Smith 2003). Vulnerability of the developing fetus to MeHg exposure was exemplified in Minamata, Japan, when pregnant women consumed seafood highly contaminated with MeHg. This resulted in extreme fetal abnormalities and neuro-toxicity (i.e., microcephaly, blindness, severe mental and physical develop-mental retardation) even among infants born to mothers with minimal symptoms (Harada 1995).

More subtle neurodevelopmental effects have been observed in populations with moderate MeHg exposures from regular consumption of fish and/or marine mammals, including associations of MeHg biomarkers at birth with decrements in memory, attention, language, and visual-motor skills in childhood (NRC 2000). Most recently, a growing body of literature has explored the impact of even lower levels of MeHg on a variety of health outcomes in both adults and children. Findings include potential adverse effects on fetal growth, neorulogic function, the cardio-vascular system, and immune function.

Given that fish is a key source of dietary protein in much of the world, MeHg contamina-tion of fish has the potential to impact the health of geographically diverse populations. Furthermore, fish is an important source of bene-ficial nutrients such as poly-unsaturated fatty acids (PUFA), iodine, selenium, and vitamin D. Development of dietary recom-menda-tions that balance nutritional benefits of fish with the contami-nant risk has been a challenge for govern-ment regulatory agencies and public health professionals (Teisl et al. 2011). In this context, charac-teriza-tion of MeHg health risks is critical for the develop-ment of optimal fish consumption guidelines (Cohen et al. 2005a; Shimshack and Ward 2010). However, there has been limited, if any, synthesis of the available literature on the health effects of low-level MeHg exposure, despite its global relevance.

To synthesize the current state of knowledge on the human health effects of low-level MeHg exposure, we focused on the epidemiologic literature of mercury concentrations measured in biologic tissue. We examined the following questions: *a*) What are the key health effects of lower, prevalent levels of MeHg exposure in the general population, and what are the strengths and limitations of recent evidence regarding those health effects? *b*) What are potential confounders or modifiers of human health risks (synergistic or antagonistic) at low-level exposure? *c*) What important gaps exist in the current literature? The ultimate goal of this review was to provide a basis for optimizing future research efforts, as well as risk–benefit assessment and exposure remediation policies, worldwide.

## Biomarkers of MeHg Exposure

Biomarkers of MeHg exposure, such as total mercury levels in hair or blood, are regarded as more accurate measures of human exposure than dietary assessment (i.e., of fish consumption) because MeHg concentrations vary both between and within fish species and because recall of specific species may be imprecise (Groth 2010). Although it is correlated with maternal hair, cord blood mercury may better reflect fetal exposure than maternal hair (Grandjean et al. 2002). Mercury is excreted in breast milk, but it is not typically used as a matrix for assessing exposure, primarily because of low concentrations and variability in the proportion present as MeHg (Björnberg et al. 2005; García-Esquinas et al. 2011; Miklavcic et al. 2011). Meconium and other tissues, such as umbilical cord, placenta, and nail tissue, although potentially useful, have not been used widely in epidemio-logic studies (Gundacker et al. 2010; Rees et al. 2007). Urinary mercury reflects inorganic mercury levels and thus is not used as an indicator of MeHg exposure; however, in hair, nails, and blood, MeHg is the primary contributor to total mercury levels (Grandjean et al. 2002).

Even the best exposure biomarkers are imprecise measures of MeHg in target organs such as the fetal brain. Furthermore, the average coefficient of variation is about 25% for cord blood mercury analysis and about twice that for maternal hair mercury (Grandjean and Budtz-Jørgensen 2010). Typically, imprecision in an exposure measure will attenuate its calculated effect (Rothman and Greenland 1998); this highlights the potential for measurement errors in MeHg exposure assessment to affect comparability of findings across studies.

## Low-Level Exposure

Because most of the published epidemiologic literature reports measures of total mercury rather than MeHg, we focused principally on studies with mercury exposure measures in blood or hair as matrices most reflective of MeHg. We excluded case reports or case series and reports that were not original research. We further limited our review to studies of low-dose mercury exposures, that is, we excluded analyses of the poisoning episodes in Japan and Iraq, as well as studies of popu-la-tions with mean measured mercury levels above any of the following: 4 µg/g in hair; 20 µg/L in cord blood, or approximately 12 µg/L in adult blood. We based our definition of low dose on a qualitative assessment of the literature and appreciation that findings from the three major cohort studies with moderate mercury exposures (the Faroe Islands, the Seychelles, and New Zealand) are already extensively reviewed (e.g., Axelrad et al. 2007; Cohen et al. 2005b; Rice 2004). Among the major prospective cohort studies of MeHg and child development in high exposure risk populations, the Faroes had the lowest mercury levels with approximately 4 µg/g in maternal hair and 20 µg/L in cord blood, on average (Steuerwald et al. 2000). We assumed a factor of 1.7 (Stern and Smith 2003) in estimating the corresponding adult blood mercury level of 12 µg/L. By design, our definition of low dose excludes studies focused on moderately MeHg-exposed groups, such as those with particularly high fish consumption (e.g., the Seychelles Islands), marine mammal consumption (e.g., the Faroe Islands and most circum-polar and other indigenous populations), or unusually contaminated fish consumption (e.g., gold-mining regions of the Amazon).

## Study Selection

Our review encompasses human epidemiologic studies that measured mercury using a biomarker. For example, in prenatal or childhood MeHg exposure assessment, studies measure primarily total mercury in whole blood (maternal, umbilical cord, or child) or hair (maternal, infant, or child). For adult exposure, MeHg levels are typically estimated using total mercury levels in whole blood, hair, or toenails. We included cohort studies irrespective of sample size and geographic location. To identify rele-vant studies, we performed a literature search for studies that analyzed the relation between mercury exposure and health outcomes using PubMed (National Library of Medicine 2012) and ScienceDirect (Elsevier 2012).

## Birth Outcomes and Infant Growth

In searching the published literature on birth outcomes and infant growth, we used the following key words: “mercury,” “infant,” “fetus,” “birth outcome,” “biomarker,” “anthropo-metric,” “maternal,” “mother,” “child,” birth,” “pregnancy,” “blood,” “cord blood,” “hair,” “birth weight,” “birth length,” “infant weight,” and “post-natal growth.” The studies reviewed are summarized in Table 1 and Supplemental Material, Table S1 (http://dx.doi.org/10.1289/ehp.1104494).

**Table 1:** Summary of studies of low-level mercury exposure.

Outcome	No. of studies	Sample size (range)	Age (range)	Exposure measures	References
Birth outcomes and infant growth										
Birth weight		8		41–645		—		Cord blood, cord tissue, maternal hair		Daniels et al. 2007; Drouillet-Pinard et al. 2010; Gundacker et al. 2010; Lederman et al. 2008; Lee et al. 2010; Lucas et al. 2004; Ramon et al. 2009; Sikorski et al.1986
Birth length		5		41–645		—		Cord blood, maternal hair		Drouillet-Pinard et al. 2010; Gundacker et al. 2010; Lederman et al. 2008; Ramon et al. 2009; Sikorski et al. 1986
Head circumference		4		41–645		—		Cord blood, maternal hair		Drouillet-Pinard et al. 2010; Gundacker et al. 2010; Lederman et al. 2008; Sikorski et al. 1986
Gestational age		5		329–1,024		—		Cord blood, cord tissue, maternal hair		Daniels et al. 2007; Drouillet-Pinard et al. 2010; Lederman et al. 2008; Lucas et al. 2004; Xue et al. 2007
Infant growth		1		921		24 months		Cord blood		Kim et al. 2011
Neurologic outcomes										
Birth–2 years		10		53–1,054		Birth–26 months		Cord blood, cord tissue, infant hair, maternal hair, maternal blood		Barbone et al. 2004; Cace et al. 2011; Cao et al. 2010; Daniels et al. 2004; Gao et al. 2007; Jedrychowski et al. 2006, 2007; Lederman et al. 2008; Oken et al. 2005; Suzuki et al. 2010
3–6 years		11		72–1,778		36 months– 6 years		Cord blood, child hair, child blood, maternal hair, maternal blood		Cao et al. 2010; Després et al. 2005; Freire et al. 2010; Ha et al. 2009; Jedrychowski et al. 2007; Lederman et al. 2008; Oken et al. 2008; Plusquellec et al. 2010; Saint-Amour et al. 2006; Stewart et al. 2003; Surkan et al. 2009
7–14 years		6		100–1,778		7–14 years		Cord blood, child hair, child blood		Boucher et al. 2010; Cao et al. 2010; Cheuk and Wong 2006; Ha et al. 2009; Surkan et al. 2009; Torrente et al. 2005
Adults		4		106–474		17 to ≥ 81 years		Adult hair, adult blood		Johansson et al. 2002; Philibert et al. 2008; Weil et al. 2005; Yokoo et al. 2003
Cardiovascular outcomes										
		8		Prospective cohort: 1,014–1,871		16–75 years		Hair, blood, toenail, urine mercury		Guallar et al. 2002; Mozaffarian et al. 2011; Rissanen et al. 2000; Salonen et al. 1995, 2000; Virtanen et al. 2005; Wennberg et al. 2011; Yoshizawa et al. 2002
				Case–control: 431–3,427 cases; 464–3,427 controls						
									
Blood pressure		1		1,240		16–49 years		Blood mercury		Valera et al. 2009; Vupputuri et al. 2005
Immunologic outcomesa										
		1		Prospective cohort: 582		29–39 months		Hair (child, maternal)		Miyake et al. 2011
		3		Cross-sectional: 61–112		Newborns		Blood (cord, maternal delivery)		Belles-Isles et al. 2002; Bilrha et al. 2003; Nyland et al. 2011a
		1		Cross-sectional: 1,990		≥ 20 years		Blood		Park and Kim 2011
See also Supplemental Material, Table S1–S4 (http://dx.doi.org/10.1289/ehp.1104494). aStudies published since the NRC report (NRC 2000).										

Overall, studies on fetal mercury exposure and birth outcomes show mixed results [see Supplemental Material, Table S1 (http://dx.doi.org/10.1289/ehp.1104494)]. A small study from Poland (*n* = 41) published in 1986 found that infant, but not maternal, hair mercury was inversely associated with birth weight without consideration of fish or seafood consumption (Sikorski et al. 1986). In a more recent, larger cohort (*n* = 554) in Spain, Ramon et al. (2009) found that cord blood mercury was inversely related to birth weight. Newborns in the highest quartile for cord blood mercury weighed 143.7 g less [95% confidence interval (CI): –2251.8, –235.6] than those in the first quartile, after adjusting for fish consumption and other variables. These authors also observed a similar pattern for small-for-gestational-age newborns, although the results were not statistically significant. In a study examining cord blood mercury and maternal blood mercury both early (12–20 weeks) and late (28–42 weeks) in pregnancy in a South Korean cohort (*n* = 417), Lee et al. (2010) observed that birth weight was inversely related to all measures of mercury exposure, with the strongest magnitude of effect observed for cord blood. Of particular interest is that the associations were more pronounced among those with the gluta-thione *S*-transferase (GST) M1 (*GSTM1*) null geno-type or both *GSTM1* and *GSTT1* null geno-types. MeHg excretion rates vary widely among individuals and involve gluta-thione conjugation by selenium-dependent GSTs (Custodio et al. 1994). Birth weight was unrelated to maternal hair mercury in a French cohort (*n* = 645) (Drouillet-Pinard et al. 2010); maternal or cord blood mercury in a New York City cohort (*n* = 329) (Lederman et al. 2008); maternal blood, hair, or cord blood mercury in a small study (*n* = 53) from Vienna, Austria (Gundacker et al. 2010); cord blood mercury in a cohort study from Nunvik, Canada (*n* = 439) (Lucas et al. 2004); and cord tissue mercury in a large study in the United Kingdom (*n* = 1,040) (Daniels et al. 2007). The French, New York City, Austrian, and U.K. studies considered fish or seafood consumption, and the Canadian study accounted for PUFA in their analysis.

We found little to no evidence of effects of low-level mercury exposure on other studied anthropometric measures at birth. Of the five studies that evaluated birth length, none found any association (Drouillet-Pinard et al. 2010; Gundacker et al. 2010; Lederman et al. 2008; Ramon et al. 2009; Sikorski et al. 1986). Likewise, four studies recorded measurements of infant head circumference at birth, but none found clear associations with mercury exposure (Drouillet-Pinard et al. 2010; Gundacker et al. 2010; Lederman et al. 2008; Sikorski et al. 1986).

Gestational age was evaluated in five studies that met our criteria. No association was observed with gestational age in the Canadian study with cord blood mercury (Lucas et al. 2004), the U.K. cohort with cord tissue mercury (Daniels et al. 2007), the New York City cohort with maternal or cord blood mercury (Lederman et al. 2008), or the French cohort with maternal hair (Drouillet-Pinard et al. 2010). In a study in Michigan (USA), however, Xue et al. (2007) found that women who delivered very preterm (< 35 weeks) were more likely to have had higher hair mercury levels (0.55–2.5 µg/g) than women who delivered at term (odds ratio = 3.0; 95% CI: 1.3, 6.7).

Of further interest, cord blood mercury (Grandjean et al. 2003; Kim et al. 2011) and late-pregnancy maternal blood mercury (Kim et al. 2011) have been associated with impaired infant growth within the first 2 years of life. One of these studies (Kim et al. 2011), based on a South Korean birth cohort, met our inclusion criteria [Table 1; see also Supplemental Material, Table S1 (http://dx.doi.org/10.1289/ehp.1104494)].

## Neurocognitive and Behavioral Outcomes

For neurodevelopmental outcomes, we searched databases using combinations of the following terms: “mercury,” “MeHg,” “blood,” “cord blood, “hair,” “low-dose,” “cognition,” “cognitive function,” “intelligence,” “IQ” (intelligence quotient), “memory,” “executive function,” “sensory function,” “visual evoked potentials,” “auditory evoked potentials,” “human behavior,” “behavior,” “neuro-behavior,” “attention,” “impulsivity,” “impulse control,” “hyperactivity,” “motor skills,” and “fine motor performance.” The studies reviewed are summarized in Tables 1 and 2 and Supplemental Material, Table S2 (http://dx.doi.org/10.1289/ehp.1104494).

**Table 2:** Summary of findings on MeHg and neurocognitive and behavioral outcomes.

Age at assessment						
Birth–2 years	3–6 years	7–14 years	Adults
Inconsistent effects: no effect; increased risk associated with prenatal or postnatal mercury		Adverse effects if adjusted for fish intake: multiple associations with prenatal mercury (psychomotor function, memory, verbal skills cognition, etc.); inconsistent effects with concurrent mercury		Inconsistent effects: protective; no effect; increased risk (e.g., electrophysiologic testing) with prenatal or postnatal mercury		Inconsistent effects: no effect or adverse neuropsychological test performance with current mercury
See also Supplemental Material, Table S2 (http://dx.doi.org/10.1289/ehp.1104494).						

Prospective cohort studies have demon-strated associations of pre-natal mercury exposure with neo-natal motor function (Suzuki et al. 2010) and behavior (Gao et al. 2007). In descriptive analyses without adjustment for potential confounders, maternal pregnancy hair mercury ≥ 1 µg/g was associated with a smaller cerebellar volume among 137 full-term Croatian newborns (Cace et al. 2011).

Among infants 6–24 months of age, prospective studies of low-level pre-natal mercury exposure have had mixed results. Mercury (adjusted for pregnancy seafood intake) was associated with decrements in infant cognition including poorer visual recognition memory at 6 months of age in U.S. infants (Oken et al. 2005) and poorer performance on both Psycho-motor Develop-ment Index (PDI) and Mental Develop-ment Index (MDI) components of the Bayley Scales of Infant Development at 12 months, as well as modest but non-significant declines at 24 months, among Polish infants in models unadjusted for fish consumption (Jedrychowski et al. 2006, 2007). In contrast, among more highly exposed urban New York City children (e.g., geometric mean cord blood mercury = 4.4 µg/L vs. 0.9 µg/L in Polish infants), no significant pre-natal mercury-associated decrements in 12- and 24-month Bayley PDI and MDI scores were observed despite adjustment for multiple potential confounders including fish consumption (Lederman et al. 2008). Similarly, in a prospective U.K. study, cord tissue mercury was not associated with scores on the MacArthur Communicative Development Inventory at 15 months of age or the Denver Developmental Screening Test at 18 months of age despite adjustment for multiple potential confounders including fish consumption (Daniels et al. 2004). However, increased measure-ment error of mercury in cord tissue (compared with hair or blood) may have contributed to null findings (Grandjean and Budtz-Jørgensen 2007; Grandjean and Herz 2011).

In preschool-age children, pre-natal mercury exposure has been consistently associated with adverse subsequent neuro-development in analyses accounting for maternal pregnancy fish consumption (Freire et al. 2010; Lederman et al. 2008; Oken et al. 2008). For example, among New York City infants in whom pre-natal mercury was not associated with significant Bayley decre-ments at 12 and 24 months of age, significant PDI (but not MDI) declines were seen at 36 months, and a 3.6-point decline in IQ (per log increase in cord blood mercury) was seen at 4 years (Lederman et al. 2008). Prenatal mercury exposures have been associated with other cognitive and psycho-motor measures in this age group, such as lower scores on tests of vocabulary (Peabody Picture Vocabulary Test) and visual-motor ability (Wide Range Assessment of Visual Motor Abilities) at 3 years of age (Oken et al. 2008) and poorer general cognition, memory, and verbal skills (McCarthy Scales of Children’s Abilities) at 4 years of age (Freire et al. 2010).

However, without adjustment for fish intake, pre-natal mercury exposures have been associated with inconsistent (Stewart et al. 2003) or even beneficial (Jedrychowski et al. 2007) findings in this age group (Stewart et al. 2003). For example, among Polish infants in whom pre-natal mercury was associated with decrements in Bayley PDI and MDI at 12 months of age (and nonsignificant decrements at 24 months), mercury was associated with non-significant increases in Bayley scores at 36 months in analyses unadjusted for fish intake (Jedrychowski et al. 2006, 2007). Among 3-year-old U.S. children born to mothers consuming MeHg-contaminated Great Lakes fish, pre-natal mercury was associated with poorer general cognition on the McCarthy Scales only among children with high pre-natal exposure to polychlorinated biphenyls (PCBs). Stewart et al. (2003) observed no mercury-related effects on the McCarthy Scales in follow up at 4.5 years of age, but their analyses were not adjusted for fish consumption.

Studies of post-natal mercury exposures in young children have produced mixed results. In a small sample (*n* = 53) of 26-month-old Italian children, higher 3-month post-partum infant or maternal hair mercury levels (e.g., < 1 µg/g vs. ≥ 1 µg/g) were marginally associated with increased risk of expected or delayed (vs. advanced) fine motor skill on the Denver Developmental Screening Test in analyses unadjusted for fish consumption (Barbone et al. 2004). Among 24-month-old U.S. participants in a randomized trial of chelation for lead poisoning, current mercury concentration was associated with better (non-significant) Bayley MDI scores (Cao et al. 2010). In follow-up of these children at 5 and 7 years of age, increased blood mercury at baseline (age 2 years) was associated with no significant differences in IQ or behavior; however, point estimates of effect were all positive (i.e., improved with increasing mercury) (Cao et al. 2010). No information about fish consumption was available in that study.

Even with adjustment for fish consumption or related nutritional measures, associations of post-natal mercury exposure with neuro-development have been inconsistent. A prospective study of Inuit children in Nunavik, Canada (the high end of the low-exposure range) is of interest given its simultaneous assessment of pre-natal and post-natal (concurrent) mercury levels and its inclusion of measures of other neuro-toxicants (PCBs, organochlorine pesticides, and lead), as well as potential nutritional confounders (selenium and omega-3 PUFA) (Després et al. 2005; Plusquellec et al. 2010; Saint-Amour et al. 2006). In a study using the Infant Behavioral Rating Scale from the Bayley Scales of Infant Development-II and scoring of video-recorded behaviors, neither pre-natal nor child-hood mercury were related to behavior at 4–6 years of age (Plusquellec et al. 2010). However, in tests of gross and fine motor skill, current, but not pre-natal, mercury was associated with increased action tremor amplitude at 4–6 years of age (Després et al. 2005). Finally, at ages 5–6 years, pre-natal mercury was associated with longer latencies in visual evoked potentials (VEPs), whereas concurrent mercury was associated with shorter VEP latencies (Saint-Amour et al. 2006), an indicator of visual information–processing efficiency. The most recent published follow-up of this population assessed performance on auditory event-related potentials (ERPs) at ages 10–13 years. Cord blood mercury was associated with both adverse and potentially beneficial effects on early auditory information processing, with increased reaction time and increased latency but fewer false alarms (i.e., false-positive errors) and greater amplitude of response on the auditory ERP task (Boucher et al. 2010). Of note, concurrent blood mercury (median, 2.8 µg/L) was not associated with auditory ERP performance.

To date, most published studies among older children of school age are cross-sectional, with mercury biomarkers indicating post-natal exposure levels. With few exceptions (Boucher et al. 2010), studies in this age group do not demon-strate adverse associations of concurrent mercury exposure with a range of cognitive and neuro-behavioral measures, and most lack information about potential confounding by fish consumption. For example, among 355 participants 6–10 years of age in a randomized trial of mercury amalgam versus composite restorations for dental carries, Surkan et al. (2009) observed no significant linear relationship of baseline mercury obtained before amalgam exposure across 18 psycho-metric tests, including measures of IQ, achievement, memory, executive function, and visual-motor and fine motor ability. Null results remained after adjustment for multiple potential confounders, including fish consumption. However, the authors identified non-linearities in dose response with suggestive evidence of improved math reasoning and visual-motor skills at hair mercury < 0.5 µg/g but decrements on the same tests at higher levels (0.5 µg/g ≤ hair mercury ≤ 1.0 µg/g) and insufficient data at hair mercury > 1.0 µg/g. In a school survey of 1,778 South Korean 6- to 10-year-old children, parental report of attention deficit hyperactivity disorder (ADHD) symptoms was unrelated to concurrent mercury (Ha et al. 2009); information about fish intake was not included in the study. Conversely, in a case–control study of ADHD among 111 7- to 8-year-old children in Hong Kong, China, higher blood mercury was associated with increased odds of ADHD diagnosis (Cheuk and Wong 2006). However, that study had a number of limitations, including lack of comparability of cases and controls on ADHD risk factors and lack of information on fish intake. Finally, in a cross-sectional assessment of 100 Spanish children 12–14 years of age, concurrent hair mercury level was correlated with better visuo-spatial skill after adjustment for age and socio-economic status (Torrente et al. 2005). These authors postulated that findings were consequent to confounding by fish consumption, which was not assessed.

Only a few cross-sectional studies of neuro-logic effects of low-level mercury exposure have been carried out in adults. Although Yokoo et al. (2003) did not include fish intake in assessments, higher hair mercury levels were associated with decrements in fine motor speed and dexterity, as well as memory and response inhibition, among 129 adults (mean age, 35 years; range, 17–81 years) from Brazilian fishing communities. Conversely among a random subset (*n* = 474) of older adults (ages 50–70 years) in the Baltimore Memory Study (Baltimore, MD), blood mercury was associated with improved manual dexterity (finger tapping) but poorer visual memory in analyses considering fish intake (Weil et al. 2005). In a study of 106 elderly Swedish adults (mean age, 87 years) by Johansson et al. (2002), blood mercury was not associated with general cogni-tive status, including memory, assessed on the Mini Mental State Examination; however, there was no assessment for confounding. Finally, among lake-fish eaters in Quebec, Canada (mean age, 47–50 years), higher hair mercury was associated with increased self-reported neuro-psychiatric symptoms of depression, anxiety, and obsessive–compulsive behavior (Philibert et al. 2008). However, this association was seen only in women, and symptom reporting was not related to blood mercury levels or serum PUFA levels.

## Cardiovascular Outcomes

For literature searches for MeHg and cardio-vascular outcomes, we used the key words “mercury” or “methylmercury,” “cardio-vascular” or “coronary,” or “hyper-tension.” References cited in articles were also identified. The studies reviewed are summarized in Tables 1 and 3 and in Supplemental Material, Table S3 (http://dx.doi.org/10.1289/ehp.1104494).

**Table 3:** Summary of findings on MeHg and cardiovascular outcomes.

Study location or group	Outcomes
Positive associations		
Finland		Hair mercury positively related to acute myocardial infarction and CHD and CVD mortality.
Europe and Israel		Toenail mercury positively associated with myocardial infarction
No associations		
Health Professionals Study and Nurses’ Health Study		Toenail mercury unrelated to incident CVD
National Health and Nutrition Examination Survey (NHANES)		Blood mercury not associated with blood pressure
Sweden		Erythrocyte mercury not associated with risk of myocardial infarction
See also Supplemental Material, Table S3 (http://dx.doi.org/10.1289/ehp.1104494).		

Although the developing brain is considered the critical target organ of MeHg toxicity for children, the cardio-vascular system may be most sensitive for adults. In the studies we reviewed, the cardio-vascular outcomes included myocardial infarction, blood pressure, heart rate variability, and athero-sclerosis. Among the studies that met our definition of a low-level exposure, the studies carried out in Finland were the first to assess the association between MeHg and cardio-vascular disease (CVD) (e.g., Salonen et al. 1995). A > 2-fold risk of acute myo-cardial infarction and mortality from coronary heart disease and CVD was associated with elevated hair mercury (> 2 µg/g). Inclusion of fatty acids had no appreciable effect on the relative risk estimates. As recently reviewed by Roman et al. (2011), subsequent studies have corroborated a potential link between MeHg and acute myocardial infarction. Mercury was associated with accelerated progression of carotid athero-sclerosis, as determined by intima-media thickness (Salonen et al. 2000). The association remained significant after adjusting for fatty acids and selenium. Rissanen et al. (2000) reported that fish oil–derived fatty acids reduced the risk of acute coronary events. In a later study of Finnish men, Virtanen et al. (2005) reported that increased mercury exposure was associated with increased risk of acute coronary events and cardio-vascular mortality, with adjustment for selenium and fatty acids. These two studies (Rissanen et al. 2000; Virtanen et al. 2005) concluded that mercury may attenuate the protective effects of fish on cardio-vascular health. A large multi-center study from Europe showed an increased risk of CVD associated with toenail mercury concentrations after controlling for DHA (docosahexaenoic acid) (Guallar et al. 2002), whereas no association was found in the U.S. Health Professionals Study, with adjustment for DHA plus eicosa-pentaenoic acid (Yoshizawa et al. 2002). In a nested case–control study combining the U.S. male health professionals and the female registered nurses cohorts (Nurses’ Health Study), Mozaffarian et al. (2011) found no adverse effects of mercury exposure on coronary heart disease, stroke, or total CVD. Findings were similar in additional analyses adjusted for DHA, eicosa-pentaenoic acid, and selenium. In a Swedish nested case–control study with low exposure, Wennberg et al. (2011) found no association between the risk of myo-cardial infarction and mercury concentration in erythro-cytes with adjustment for DHA plus eicosa-pentaenoic acid. Another nested case–control study reported an inverse association between myo-cardial infarction and erythro-cyte mercury (Hallgren et al. 2001); however, that study did not meet our definition of low-level exposure.

Several studies have found an association between increased mercury and increased blood pressure in adults, although only two met our low-dose exposure definition: Valera et al. (2009) adjusted for fish nutrients (DHA, eicosa-pentaenoic acid, and selenium), whereas Vupputuri et al. (2005) controlled for fish intake. In cross-sectional population data from the U.S. National Health and Nutrition Examination Survey (NHANES), associations were seen only among individuals who did not consume fish (Vupputuri et al. 2005). Among children more heavily exposed than criteria used in our review, an association between pre-natal mercury exposure and childhood blood pressure has been observed in some (Sorensen et al. 1999; Thurston et al. 2007) but not all (Grandjean et al. 2004) studies; however, information on nutrients and fish consumption was not available in these studies.

MeHg may induce oxidative stress, an early biological response that can produce cell damage in several organs or systems including the cardio-vascular system (Grotto et al. 2009). Experimental models suggest that oxidative stress plays an important role in the toxico-dynamics of mercury (Grotto et al. 2010). A few recent studies have examined associations between mercury exposure and oxidative stress or anti-oxidant defense in populations exposed through fish consumption, although the findings have been inconclusive (Bélanger et al. 2008; Grotto et al. 2010; Pinheiro et al. 2008). These studies, however, have reported mercury concentrations that exceeded our definition of low-level exposure. Except for Grotto et al. (2010), information on fish intake was not available.

## Immunologic Outcomes

In 2000, the NRC summarized the available literature on immuno-toxicity of mercury (NRC 2000). Study results showed that occupational exposure to inorganic forms of mercury was associated with alterations in B lymphocytes, T-helper cells, T-suppressor cells, and T-cell proliferative responses (Moszczynski et al. 1995; Queiroz and Dantas 1997a, 1997b). The NRC report also cited several animal studies involving exposure to MeHg and indicators of immuno-toxicity (e.g., Ilbäck et al. 1991). The NRC (2000) concluded that the immune system appears to be sensitive to mercury and noted that toxicologic studies have observed effects on immune-cell ratios, cellular response, and the developing immune system. However, at the time of the NRC report, there were no published epidemiologic studies of MeHg and immune function.

For immunologic outcomes, we focused on studies published since the comprehensive NRC report (i.e., post-1999), most of which have hair or blood mercury levels well in excess of the low-level range of our review or focus on elemental mercury exposure. For example, evidence of mercury-associated immuno-toxicity, including increased frequency of anti-nuclear auto-antibodies, changes in serum cytokine levels, and risk of malaria infection, has been observed in studies of heavily exposed Amazonian fish eaters and gold-mining populations (Alves et al. 2006; Crompton et al. 2002; Gardner et al. 2010; Nyland et al. 2011b). However, urinary mercury levels reflective of elemental mercury exposure from amalgam dental restorations in children has not been associated with immuno-toxicity (Shenker et al. 2008).

Table 1 and Supplemental Material, Table S4 (http://dx.doi.org/10.1289/ehp.1104494) summarize the studies with low-level MeHg exposures that we reviewed. Nyland et al. (2011a) reported signifi-cant correla-tions of both maternal and cord blood mercury (respective geometric means of 6.9 µg/L and 9.6 µg/L) with increases in cord blood total IgG among 61 mother–infant pairs in the Brazilian Amazon. In that study, which did not adjust for fish consumption, blood mercury was not associated with either maternal or fetal levels of anti-nuclear auto-antibodies or serum cyto-kines. In a recent population-based survey of Korean adults (*n* = 1,990), higher blood mercury (geometric mean, 4.3 µg/L) was associated with increased risk of self-reported atopic dermatitis in multi-variable analyses adjusted for fish consumption (Park and Kim 2011). As part of the Osaka Maternal and Child Health Study, Miyake et al. (2011) evaluated 582 mother–child pairs in Japan for mercury exposure, using both maternal hair (median, 1.5 µg/g) and hair collected from their offspring 29–39 months of age (median, 1.4 µg/g). After adjustment for multiple potential confounders, including maternal fish consumption during pregnancy and child fish consumption, the authors detected no association between either maternal or child hair mercury and risk of childhood wheeze or eczema.

Belles-Isles et al. (2002) compared Canadian infants (*n* = 48) born to a population of subsistence fishers with a reference population (*n* = 60 infants) from coastal towns (geometric mean cord blood mercury of 1.8 and 0.9 µg/L, respectively). Cord blood mercury was inversely correlated with the proportion of naive helper T cells and plasma IgM levels in cord blood but unrelated to multiple other measures of T-, B-, and natural killer cell proportions and function. Of note, these analy-ses were not adjusted for potential confounding, including the substantial organo-chlorine exposures and greater prevalence of smoking during pregnancy among subsistence fishers compared with referent mothers. Bilrha et al. (2003) studied children born to subsistence fishers (*n* = 47) and town residents (*n* = 65) from the same Canadian region to expand on assessments in Belles-Isles et al. (2002). In correlational analyses (unadjusted for potential confounders), the authors observed no relationship between cord blood mercury and cord blood lymphocyte activation markers or cytokine secretion.

## Summary

*Birth outcomes and infant growth.* To date, relatively few studies have evaluated the effects of MeHg on fetal growth and gestation. An advantage of studying birth outcomes is the limited time window of exposure. Lee et al. (2010) measured maternal mercury levels at multiple time points during pregnancy, with similar results. The largest number of studies assessed birth weight, but the differing matrices used to measure exposure make comparison between studies challenging. Several studies evaluated cord blood mercury concentrations but employed differing statistical approaches (e.g., analysis of categorical vs. continuous data). Of interest, two studies that provided a statistical estimate adjusted for fish intake both found evidence of reduced birth weight in relation to cord blood mercury concentrations (Lee et al. 2010; Ramon et al. 2009), whereas another study that did not adjust for fish and seafood intake (but did examine plasma PUFA) did not observe such associations (Lucas et al. 2004). Of the studies that evaluated associations of cord blood mercury levels with gestational age, one provided a statistical estimate of an association with prematurity (Xue et al. 2007); another study did not find a relation with gestational age but did not specifically evaluate preterm deliveries (Lucas et al. 2004). Size for gestational age is of interest, but only one study to date has reported on this outcome (Ramon et al. 2009); although results were suggestive of a mercury effect, they lacked statistical power. Thus, the potential impact of low-level MeHg on fetal growth is uncertain, although there is suggestive evidence of an effect. Finally, in addition to possible influences of low-level *in utero* mercury exposure on fetal growth, recent data raise the possibility of effects on post-natal growth (Kim et al. 2011).

*Neurocognitive and behavioral outcomes.* The literature on low-level mercury and neuro-development underscores the importance of exposure timing, precision of the exposure assessment, confounding, age at assessment, the specific neuro-behavioral outcome, sex differences, and dose–response modeling in determining the observed results. For example, in children, pre-natal exposure may be more deleterious than post-natal exposure for most, but likely not all, neuro-developmental measures (Myers et al. 2009; Saint-Amour et al. 2006). With some exceptions (Daniels et al. 2004; Plusquellec et al. 2010), studies in which null, or potentially beneficial, associations with mercury were seen typically lacked measures of fish consumption or related nutrients, such as PUFA, which could confound findings and account for null or apparently neuro-protective mercury effects (Cao et al. 2010; Ha et al. 2009; Johansson et al. 2002; Torrente et al. 2005). An example of the complexity of this literature is the variability among associations of low-level pre-natal mercury with performance on the Bayley Scales of Infant Development between 12 and 36 months of age. Prenatal mercury effects were seen only at 12 months of age in some populations (Jedrychowski et al. 2006, 2007) or only at 36 months of age in others (Lederman et al. 2008). In contrast, post-natal exposure has been associated with improved Bayley performance at 24 months of age (Cao et al. 2010) but without adjustment for fish consumption. In the low-level exposure literature, differences in exposure and confounding (including diet), as well as differences in neuro-developmental test sensitivity, may account for some apparent inconsistencies.

Findings in children and adults are difficult to compare, at least in part, because of differences in testing protocols. In addition, among populations with chronic mercury exposure, associations with mercury measured in later life may reflect the long-term developmental consequences of early-life exposure. However, where there is general overlap in assessments, findings are surprisingly concordant. For example, concurrent blood mercury was associated with increased action tremor amplitude in 4- to 6-year-old Inuit children (Després et al. 2005) and poorer fine motor speed and dexterity in Brazilian adults (Yokoo et al. 2003). Regardless of age, certain domains of function may be more sensitive to mercury toxicities, including memory (Freire et al. 2010; Oken et al. 2005; Weil et al. 2005), verbal or language skills (Freire et al. 2010; Lederman et al. 2008; Oken et al. 2008), and visual-motor functions (Oken et al. 2008; Surkan et al. 2009). In contrast, except in a case–control analysis of ADHD (Cheuk and Wong 2006), adverse behaviors were not associated with mercury exposure in children (Cao et al. 2010; Ha et al. 2009; Plusquellec et al. 2010).

*Cardiovascular outcomes.* No clear pattern has been observed among the limited number of studies that assessed the association between low-level mercury and cardio-vascular function. The evidence, however, suggests that adverse cardio-vascular effects can occur at very low levels of mercury exposure. For example, men who had a hair mercury concentration of ≥ 2 µg/g had a 2-fold increased risk of suffering an acute myo-cardial infarction or dying from coronary heart disease or CVD compared with less-exposed men (Salonen et al. 1995). Although essential fatty acids from fish may reduce the risk of acute coronary events, mercury in fish could attenuate this bene-ficial effect (Rissanen et al. 2000). Eastern Finnish men in the lowest two tertiles of hair mercury concentration (0–2 µg/g) who also were in the highest quintile of serum fatty acid levels had a 67% reduced risk of acute coronary events compared with men in the highest tertile of hair mercury who were in the lowest quintile of serum fatty acids. In that cohort, the increased risk seemed to occur at hair mercury concentrations > 2 µg/g, that is, only 2 times the level corresponding to daily mercury intake at the U.S. EPA reference dose (a level estimated to be without significant risk of adverse effects over a lifetime) (Salonen et al. 1995). In contrast, in a large multi-center cohort in Europe, an increased risk of myo-cardial infarction was found in participants with toenail mercury concentrations of 0.36 µg/g, approximating an even lower hair mercury level of < 1 µg/g (Guallar et al. 2002; Ohno et al. 2007).

*Immunologic outcomes.* Relatively high exposure to elemental mercury has been linked to a range of immuno-logic outcomes (including markers of auto-immunity) in epidemiologic studies, but evidence of immuno-toxicity of low-level MeHg is inconclusive in the limited literature available to date. It is difficult, however, to make any definitive statements as to which forms of mercury are likely to affect which immune components. Much of the available epidemiologic literature is limited by small sample sizes, incomplete adjustment for potential confounders, and lack of consistency across exposure media (e.g., only hair in some populations, only urine in others), which have impeded making the comparisons across studies that are necessary for refined hypothe-sis generation and testing. Dialogue among mercury researchers is also needed to identify optimal measures of potential immune impairment.

## Priorities for Future Studies

Despite evidence of possible differences in mercury toxicities between the sexes (Marsh 1994), most studies did not report assessing such differences. Only two of the reviewed studies on neuro-logic outcomes reported sex differences in mercury effects: Only male infants had mercury-associated decrements in behavior (Gao et al. 2007), and only adult women reported increased psychiatric symptoms associated with mercury (Philibert et al. 2008). In addition to sex-specific effects, other host factors could influence susceptibility to MeHg effects. Although research on these factors is scarce, a recent Korean study of fetal growth found evidence of genetic susceptibility, with genetic variation in *GSTM1* and *GSTT1* affecting risk (Lee et al. 2010). Future studies should emphasize the use of the most precise exposure indicators in sensitivity analyses to model the impact of likely imprecision (Budtz-Jørgensen et al. 2003). Similarly, future studies should use outcome measures for which there are mechanistic or other *a priori* bases for assuming mercury sensitivity. The potential for non-linear dose–response relationships (e.g., a threshold dose response) needs to be considered consistently. Because MeHg originates from fish and seafood, which also contain nutrients that may be bene-ficial to health (including birth outcomes, neuro-development, cardio-vascular health, and immune function), proper adjustment for potential negative confounding by nutrition is crucial in any future study.

## Summary of Findings

The possibility that MeHg at low exposure levels might affect fetal growth among susceptible sub-groups and infant growth requires further investigation.

There is evidence that low levels of pre-natal MeHg exposure may cause early childhood neuro-cognitive effects. The possibility of non-linear effects, as well as possible differential effects by sex, should be evaluated for older children and adults.

There are no clear patterns across populations or for specific study end points for cardio-vascular effects of low-level MeHg exposure. Future studies targeted at mechanisms of effects may be informative (e.g., effects on heart rate variability). In addition, sexual dimorphism and non-linearity should be considered.

Although there are some indications of MeHg-associated immune effects, epidemiologic studies addressing this question are scarce, have small sample sizes, and include limited assessment of important potential confounders. Thus, we cannot draw any conclusions at this time.

## Supplemental Material

(438 KB) PDF
