# Supplementary material for: Evidence on the Human Health Effects of Low-Level Methylmercury Exposure
Source: Environ Health Perspect. 2012 Jan 24;120(6):799–806. doi: 10.1289/ehp.1104494 (PMC3385440; doi:10.1289/ehp.1104494)
Supplement: (438 KB) PDF [file ehp.1104494.s001.pdf]

Supplemental Material

Evidence on the Human Health Effects of Low Level Methylmercury Exposure

Margaret R. Karagas, Anna L. Choi, Emily Oken, Milena Horvat, Rita Schoeny, Elizabeth Kamai, Whitney Cowell, Philippe Grandjean, Susan Korrick

Table of Contents:

Supplemental Material, Table S1. Published Studies of Low-Level Methylmercury Exposure and Birth Outcomes ……………………………………………………………………….pg. S3 – S5

Supplemental Material, Table S2. Published Studies of Low-Level Methylmercury Exposure and Neurocognitive and Behavioral Outcomes ……………………………………………pg. S6 – S13

Supplemental Material, Table S3. Published Studies of Low-Level Methylmercury Exposure and Cardiovascular Outcomes ……………………………………………………………pg. S14 – S16

Supplemental Material, Table S4. Published Studies of Low-Level Methylmercury Exposure and Immunologic Outcomes ……………………………………..……………………pg. S17 – S18

References ……………………………………………………………………………pg. S19 – S23

Supplemental Material, Table S1. Published Studies of Low-Level Methylmercury Exposure and Birth Outcomes and Infant Growth (listed by year of publication)

Author	Population/ Study Group	Study Design	Sample Size	Exposure Assessment	Exposure Level	Outcomes	Findings	Other contaminants/ nutrients examined
Sikorski et al., 1986	Lublin, Poland	Birth Cohort, enrolled at labor/delivery	N=41	Maternal scalp hair Hg	Mean: 1.88 µg/g<br>Range: 0.02-40.60 µg/g	Birth weight, birth length, head and chest circumference	Significant inverse correlation between Hg in infant hair and birth weight	None
				Maternal pubic hair Hg	Mean: 1.01 µg/g<br>Range: ND-31.86 µg/g			
				Infant hair Hg	Mean: 0.11 µg/g<br>Range: ND-0.62 µg/g			
Lucas et al., 2004	Nunavik, Canada	Birth Cohort, enrolled at labor/ delivery	N=439	Cord blood Hg	Geomean: 14.1 µg/L (95% CI 13.,15.2)	Birth weight, gestational age	Mercury levels not significantly associated with birth weight or gestational age in unadjusted models	Cord plasma concentration of n-3 PUFA, PCB
Daniels et al., 2007	Bristol, England and surrounding areas	Birth Cohort, enrolled during prenatal visits	N=1040	Cord tissue Hg	Median: 0.01 µg/g wet weight	Birth weight, gestational age	Hg in cord tissue not associated with gestational age or birth weight	Fish intake
Xue et al., 2007	Five Michigan communities (rural, suburban, urban)	Birth Cohort, enrolled at 15th-27th week of pregnancy	N=1024	Maternal hair Hg	Mean: 0.29 µg/g<br>Median: 0.23 µg/g<br>Range: 0.01-2.50 µg/g<br>highest Hg levels ($\geq 90^{th}$ percentile, 0.55-2.50 µg/g)	Gestational age	Women who delivered at <35 weeks (very pre-term) were more likely to have had hair Hg levels $\geq 90^{th}$ percentile (.55-2.5 µg/L).<br>Hg levels not associated with delivery at 35-36 weeks	Fish consumption
Lederman et al., 2008	NYC hospitals close to Ground Zero, deliveries between 12/12/01 and 6/26/02	Birth Cohort, enrolled at labor/ delivery	N=329	Maternal whole blood Hg	Geomean: 1.6 µg/L; (95% CI 1.4,1.81)	Birth weight, birth length, head circumference, gestational age	No significant relationship between Hg and birth outcomes.	Seafood consumption
				Cord blood Hg	Geomean: 4.44 µg/L; (95% CI 3.91, 5.04)			

Supplemental Material, Table S1 (Cont.) Published Studies of Low-Level Methylmercury Exposure and Birth Outcomes and Infant Growth (listed by year of publication)								
Author	Population/ Study Group	Study Design	Sample Size	Exposure Assessment	Exposure Level	Outcomes	Findings	Other contaminants/ nutrients examined
Ramon et al., 2009	Hospital La Fe in Valencia, Spain	Birth Cohort, enrolled at 10th-13th week of pregnancy	N=554	Cord blood Hg	Geomean: 9.4 µg/L (95% CI 8.8, 10.2)	Birth weight, birth length, gestational age	Increased cord blood Hg associated with reduced birth weight and increased risk of being born small for gestational age (SGA) for length.<br>Large oily fish associated with higher risk for SGA for weight.<br>Canned tuna consumption associated with higher birth weight.<br>Lean fish consumption associated with lower risk of SGA for length.	Adjusted by type of fish consumed
Lee et al., 2010	Seoul, Cheonan, and Ulsan, South Korea	Birth Cohort, enrolled in first trimester of pregnancy	N=417	Cord blood Hg	Geomean: 5.53 µg/L<br>Range 0.23-24.1 µg/L	Birth weight	Lower birth weight with GSTM1 null type and increasing cord blood Hg, and GSTT1 null type and increasing Hg in late pregnancy.<br>No association with GSTM1/GSTT1 present types.<br>>90th percentile late pregnancy Hg levels associated with lower birth weight, especially when GSTM1/ GSTT1 double null	Fish intake
				Early pregnancy (12-20 wks) maternal whole blood Hg	Geomean: 3.67 µg/L<br>Range: 0.27-22.6 µg/L			
				Late pregnancy (28-42 wks) maternal whole blood Hg	Geomean: 3.30 µg/L<br>Range: .12-18.5 µg/L			

Supplemental Material, Table S1 (Cont.) Published Studies of Low-Level Methylmercury Exposure and Birth Outcomes and Infant Growth (listed by year of publication)

Author	Population/ Study Group	Study Design	Sample Size	Exposure Assessment	Exposure Level	Outcomes	Findings	Other contaminants/ nutrients examined
Drouillet-Pinard et al., 2010	France	Birth Cohort, enrolled before 24th week of pregnancy	N=645	Maternal hair Hg	Median: 0.52 µg/g<br>IQR: 0.30-0.82 µg/g<br>SD.: 2.6	Ultrasound measures; birth weight, birth length, head circumference, sum of skin folds, gestational length, placental weight	No consistent association between Hg level and fetal growth.	Seafood consumption, Se levels
Gundacker et al., 2010	Vienna, Austria	Birth Cohort, enrolled during second trimester of pregnancy	N=53	Maternal whole blood T-Hg	Range: 0.1-5.2 µg/L	Birth weight, birth length, head circumference at birth	Maternal hair Hg significantly associated with birth length in bivariate analyses only; otherwise Hg levels not associated with newborn anthropometry	Pb, seafood consumption
				Placenta T-Hg	Range: 0.1-11.7 µg/g			
				Placenta I-Hg	Range: 0.1-4.3 µg/g			
				Placenta Me-Hg	Range: 0.1-9.2 µg/g			
				Cord blood T-Hg	Range: 0.2-6.8 µg/L			
				Meconium T-Hg	Range: 0.4-128 µg/g			
				Breast milk I-Hg	Range: 0.1-2.0 µg/L			
				Maternal hair T-Hg	Range: 0.05-0.77 µg/g			
Kim et al., 2011	Seoul, Cheonan, and Ulsan, South Korea	Birth Cohort, enrolled in first trimester of pregnancy	N=797	Cord blood Hg	Geomean: 5.52 µg/L<br>SD: 1.6 µg/L	Infant weight at 6, 12, and 24 months of age	Inverse relationship between infant weight at 24 months and cord blood and late pregnancy maternal blood Hg levels<br>No significant inverse relationship between infant weight at 12 months and Hg level	Fish intake
				Early pregnancy (12-20 wks) maternal whole blood Hg	Geomean: 3.4 µg/L<br>SD: 1.6 µg/L			
				Late pregnancy (28-42 wks) maternal whole blood Hg	Geomean: 3.1 µg/L<br>SD: 1.7 µg/L			

Supplemental Material, Table 1 Abbreviations: CI (confidence interval); Geomean (geometric mean); GSTM1 (Glutathione-*S*-transferases M1); GSTT1 (Glutathione-*S*-transferases T1); Hg (mercury); I-Hg (inorganic mercury); IQR (interquartile range); Me-Hg (methylmercury); n-3 PUFA (omega-3 polyunsaturated fatty acid); ND (non-detect); PCBs (polychlorinated biphenys); SD (standard deviation); Se (selenium); T-Hg (total mercury);

Supplemental Material, Table S2. Published Studies of Low-Level Methylmerucy Exposure and Neurocognitive and Behavioral Outcomes (listed by exam age)

Author	Exam Age	Population/Study Group	Study Design	Mercury Exposure Assessment	Outcomes (analyses)	Findings	Comments
Suzuki et al., 2010	3 days	498 mother-newborn (term) pairs in Tohoku, Japan	Prospective birth cohort	Median (range): Maternal hair 2.0 (0.3-9.4) µg/g	Neonatal Behavioral Assessment Scale (NBAS); (multivariable linear regression)	0.12 point ↓motor cluster per log increase hair Hg	Models adjusted for multiple confounders including PCBs & seafood consumption
Gao et al., 2007	3 days	384 mother-newborn (term) pairs in Zhoushan City, China	Prospective birth cohort	Geomean (IQR): Cord blood 5.6 (4.0-7.8) µg/L Maternal hair 1.2 (0.9-1.7) µg/g	Neonatal Behavioral Neurological Assessment (NBNA); (logistic regression stratified by sex	↑ prenatal Hg → ↓ NBNA behavior score in males: OR = 1.2, 95% CI 1.1 to 1.4 per log increase cord blood Hg	Limited assessment for confounding; multiple (6) outcomes; results attenuated using maternal hair Hg levels
Cace et al., 2011	Neonatal	137 mother-newborn pairs in Croatia	Prospective pregnancy cohort	Mean (range): Maternal hair 0.9 (0.02-8.7) µg/g	Neurosonographic exam (compare median cerebellar dimensions for high vs. low exposed)	↓1.6 mm (average) cerebellar length where hair Hg ≥ 1 µg/g (n=30, mean Hg 2.4 µg/g)	Descriptive analysis only; no modeling, no adjustment for confounders, minimal information re. population.
Oken at al., 2005	6 mos	135 mother-infant pairs in Massachusetts, U.S.A.	Prospective pregnancy cohort	Mean (range): Maternal hair @ parturition: 0.6 (0.02-2.4) µg/g	Visual Recognition Memory (VRM); (multivariable linear regression)	↑ prenatal Hg → ↓ VRM score: -7.5 pts, 95% CI -13.7 to -1.2 per ppm hair Hg	Models adjusted for multiple confounders including pregnancy fish consumption
Jedrychowski et al., 2006	12 mos	233 mother (non-smoking)-infant pairs in Krakow, Poland	Prospective pregnancy cohort	Geomean (range): Maternal blood @ birth: 0.6 (0.1-3.4) µg/L Cord blood: 0.9 (0.1-5.0) µg/L	Bayley Scales of Infant Development (BSID-II); (multivariable logistic regression; outcome = performance on both Psychomotor Developmental Index (PDI) and Mental Developmental Index (MDI))	↑ prenatal Hg → ↑risk of delay on PDI or MDI (n=36): RR = 3.6, 95% CI 1.4 to 9.1 for cord blood Hg > 0.8 µg/L RR = 2.8, 95% CI 1.2 to 6.8 for maternal blood Hg > 0.5 µg/L	Limited assessment for confounding (e.g., ascertained fish intake but did not include in models)

Supplemental Material, Table S2 (Cont.) Published Studies of Low-Level Methylmerucy Exposure and Neurocognitive and Behavioral Outcomes (listed by exam age)							
Author	Exam Age	Population/Study Group	Study Design	Mercury Exposure Assessment	Outcomes (analyses)	Findings	Comments
Jedrychowski et al., 2007	12, 24, 36 mos	374 mother (non-smoking)-infant pairs in Krakow, Poland	Prospective pregnancy cohort	(see Jedrychowski et al., 2006)<br>High exposure: Cord blood > 0.9 µg/L (n=177)<br>Low exposure: Cord blood ≤ 0.9 µg/L (n=197)	Bayley Scales of Infant Development (BSID-II); (multivariate linear regression; Generalized Estimating Equations (GEE))	↑ prenatal Hg → ↓ PDI (cord Hg >0.9 vs. ≤ 0.9 µg/L)<br>-2.3 pts (p=0.04) at 12 mos<br>-1.4 pts (p=0.08) GEE model 12-36 mos<br>↑ prenatal Hg → ↓ MDI (cord Hg > 0.9 vs. ≤ 0.9 µg/L)<br>-2.8 pts (p=0.01) at 12 mos<br>-1.4 pts (p=0.11) GEE model 12-36 mos<br><br>Null PDI at 24, 36 mos (cord Hg > 0.9 vs. ≤ 0.9 µg/L):<br>-1.4 pts (p=0.20) at 24 mos<br>+1.2 pts (p=0.37) at 36 mos<br><br>Null MDI at 24, 36 mos (cord Hg > 0.9 vs. ≤ 0.9 µg/L):<br>-1.1pts (p=0.42) at 24 mos<br>+1.1pts (p=0.37) at 36 mos	Ascertained fish intake but did not include in models. Children lost to follow up at 24 or 36 mos did more poorly on Bayley than those retained.

Supplemental Material, Table S2 (Cont.) Published Studies of Low-Level Methylmerucy Exposure and Neurocognitive and Behavioral Outcomes (listed by exam age)

Author	Exam Age	Population/Study Group	Study Design	Mercury Exposure Assessment	Outcomes (analyses)	Findings	Comments
Daniels et al., 2004	15, 18 mos	1054 children in Briston, UK (subset from the Avon Longitudinal Study of Parents & Children (ALSPAC))	Prospective pregnancy cohort	Median (IQR): Cord tissue 0.01 (0.008-0.02) µg/g wet weight	MacArthur Communicative Development Inventory (MCDI) at 15 mos; Denver Developmental Screening Test (DDST) at 18 mos. (Generalized Linear Models, GLM)	No association of cord tissue Hg with MCDI or DDST (language & communication skills); maternal & infant fish consumption → ↑MCDI & ↑DDST scores	Models adjusted for multiple confounders including fish intake; cord tissue an unusual matrix, (levels ~25% of Faroes cord tissue) with potential greater Hg measurement error than other matrices.
Barbone et al., 2004	median 26 mos (range 18-30)	53 children in northeastern Italy (coastal & inland communities)	Prospective cohort	Mean MeHg ~3 mos post-partum (advanced vs. age-appropriate or delayed fine motor): Maternal hair 0.6 vs. 1.0 µg/g Infant hair 0.5 vs. 0.7 µg/g	Denver Developmental Screening Test (DDST-II) once between 18-30 mos.	↑ postnatal Hg → ↑risk of expected or delayed DDST fine motor-adaptive skill RR = 1.5, 95% CI 1.0 to 2.1 (maternal hair MeHg ≥1 vs. <1 µg/g)	Limited assessment for confounding (univariate analyses, fish intake assessed but not modeled). No prenatal exposure measure. Small n & low participation (original cohort n=243)
Lederman et al., 2008	12, 24, 36, 48 mos	329 mother (non-smoking)-infant pairs; women pregnant during 2001 U.S. World Trade Center attack & living or working in area	Prospective birth cohort	Geomean (range): Cord blood 4.4 (0.1-63) µg/L Maternal peripartum blood 1.6 (0.07-16) µg/L	Bayley Scales of Infant Development (BSID-II) at 12, 24, 36 mos; Wechsler Preschool & Primary Scale of Intelligence (WPPSI-R) at 48 mos (multiple linear regression)	At 12, 24 mos (n=130-132): Non-sig PDI, MDI decreases per log cord blood Hg<br><br>At 36 mos (n=111): ↓4.1 pts PDI per log cord blood Hg (non-sig MDI decreases)<br><br>At 48 mos (n=107): ↓3.2 pts performance IQ per log cord blood Hg ↓2.9 pts verbal IQ per log cord blood Hg ↓3.6 pts full scale IQ per log cord blood Hg	Models adjusted for multiple confounders including fish intake. Except full scale IQ, Hg associations with other outcomes only significant when models include fish consumption.

Supplemental Material, Table S2 (Cont.) Published Studies of Low-Level Methylmerucy Exposure and Neurocognitive and Behavioral Outcomes (listed by exam age)							
Author	Exam Age	Population/Study Group	Study Design	Mercury Exposure Assessment	Outcomes (analyses)	Findings	Comments
Stewart et al., 2003	38, 54 mos	212 mother (including Lake Ontario contaminated fish consumers)-infant pairs	Prospective pregnancy cohort	Median (IQR): Maternal hair (1st half preg) 0.5 (0.4-0.6) μg/g (2nd half preg) 0.5 (0.4-0.7) μg/g	McCarthy Scales of Children's Abilities (MSCA); (linear regression)	At 38 mos: ↓0.3 pts General Cognitive Index (GCI) per unit increase hair Hg where high PCB levels (n=55)<br><br>At 54 mos: No relation of Hg with MSCA	Models adjusted for multiple confounders including other neurotoxicants (e.g., PCBs). No main effect of Hg; effect only seen in context of high PCBs but small n.
Oken et al., 2008	3 yrs	341 mother-infant pairs in Massachusetts, U.S.A.	Prospective pregnancy cohort	Mean (range): Pregnancy (RBC): (0.03-21.9) ng/g	Peabody Picture Vocabulary Test (PPVT) & Wide Range Assessment of Visual Motor Abilities (WRAVMA); (multivariable linear regression)	For upper decile (n=35) vs. <90th percentile RBC Hg:<br>PPVT: ↓4.5 pts, 95% CI -8.5 to -0.4<br>WRAVMA: ↓6.0 pts, 95% CI -10.9 to -1.1 matching score; ↓4.6 pts, 95% CI -8.3 to -0.9 total score	Models adjusted for multiple confounders including fish intake & n-3 PUFA. RBCs an unconventional matrix (estimate 90th percentile approximates 1 μg/g hair Hg)
Freire et al., 2010	4 yrs	72 mother-son pairs in Granada, Spain	Prospective birth cohort (cross-sectional analysis)	Geomean: Child hair at test 0.96 μg/g 95% CI 0.8-1.2 μg/g	McCarthy Scales of Children's Abilities (MSCA); (multivariable linear regression)	Child hair Hg ≥ 1 μg/L (vs. < 1 μg/L):<br>↓6.6 pts, 95% CI -13.0 to -0.2 gen. cognitive score; ↓8.4 pts, 95% CI -16.0 to -0.8 memory score; ↓ 7.5 pts, 95% CI -15.0 to -0.02 verbal score	Models adjusted for multiple confounders including fish intake. Effect of fish intake varied by type (total fish intake mostly adverse). All male cohort. Study population small subset of overall cohort (n~700)

Supplemental Material, Table S2 (Cont.) Published Studies of Low-Level Methylmerucy Exposure and Neurocognitive and Behavioral Outcomes (listed by exam age)							
Author	Exam Age	Population/Study Group	Study Design	Mercury Exposure Assessment	Outcomes (analyses)	Findings	Comments
Despres et al., 2005	4-6 yrs	110 Inuit children in Nunavik, Canada (follow up of Cord Blood Monitoring Program, n=483)	Prospective birth cohort	Geomean (range): Cord blood 15.9 (1.8-104) µg/L Child blood at test: 5.9 (0.2-38.2) µg/L	Multiple neuromotor measures (gross & fine motor, reaction time); (hierarchical multivariate linear regression)	↑blood Hg at test → ↑action tremor amplitude (no relationship with prenatal Hg)	Models assessed multiple confounders including other neurotoxicants (PCBs, Pb, organochlorine pesticides) & nutrients (Se, n-3 PUFAs)
Plusquellec et al., 2010	4-6 yrs	110 Inuit children in Nunavik, Canada (follow up of Cord Blood Monitoring Program, n=483)	Prospective birth cohort	Mean (range): Cord blood 22.2 (1.8-104) µg/L Child blood at test 9.6 (0.2-38.2) µg/L	Infant Behavioral Rating Scale (from Bayley Scales of Infant Development); Coded behavior from video recordings of fine motor testing; (multivariate linear regression)	No Hg-child behavior associations	Models assessed multiple confounders including other neurotoxicants (PCBs, Pb, organochlorine pesticides) & nutrients (Se, n-3 PUFAs)
Saint-Amour et al., 2006	5-6 yrs	102 Inuit children in Nunavik, Canada (follow up of Cord Blood Monitoring Program, n=483)	Prospective birth cohort	Geomean (range): Cord blood 16.5 (1.8-104) µg/L Child blood at test: 5.9 (0.2-38.2) µg/L	Visual Evoked Potentials (VEPs); (multivariable linear regression)	↓3-4 ms latency[1] per log ↑child blood Hg<br>↑3 ms latency[1] per log ↑cord blood Hg<br>[*](time from visual stimulus onset to wave peak)	Models assessed multiple confounders including PCBs, Se & n-3 PUFAs& their interaction with exposure. Small #observations (n=69-72) in final analyses

Supplemental Material, Table S2 (Cont.) Published Studies of Low-Level Methylmerucy Exposure and Neurocognitive and Behavioral Outcomes (listed by exam age)							
Author	Exam Age	Population/Study Group	Study Design	Mercury Exposure Assessment	Outcomes (analyses)	Findings	Comments
Cao et al., 2010	2, 5, 7 yrs	780 children from urban Ohio, Pennsylvania, Maryland, New Jersey, U.S.A.	Randomized clinical trial of succimer Rx for moderate childhood lead poisoning	Median (IQR): Child blood at 2 yr baseline 0.6 (0.4-0.8) µg/L Child blood post treatment 0.5 (0.4-0.8) µg/L	Bayley Scales of Infant Development (BSID-II) at age 2 yrs; Multiple cognitive & behavioral assessments at ages 5 & 7; (general linear models, GLM)	Per log child blood MeHg at 2 yr baseline:<br>↑0.3 pts, 95% CI -1.3 to 1.9 Mental Dev Index (sig where high BPb)<br>↑0.8 pts, 95% CI -0.7 to 2.3 IQ at 5 yrs<br>↑0.5 pts, 95% CI -1.0 to 2.1 IQ at 7 yrs<br><br>Behavior (also non-sig, improved pt estimates).	Models adjusted for multiple confounders including BPb & treatment group. No information about fish consumption.
Cheuk & Wong, 2006	Mean (SD) cases 7.1 (2.5) controls 7.8 (3.5) yrs	52 ADHD cases (from referral clinic) 59 controls (hospitalized for acute upper resp infection) in Hong Kong, China	Case-control study	Geomean (95% CI): Case blood 3.6 (3.1-4.3) µg/L Ctrl blood 2.3 (2.0-2.7) µg/L	Clinical diagnosis of ADHD (multivariate logistic regression)	Child blood Hg > 5.8 µg/L vs. ≤ 5.8 µg/L:<br>OR = 9.7, 95% CI 2.6 to 36.5 for ADHD diagnosis	No information about fish consumption but assumed relatively high. Ctrls were not healthy; cases & ctrls differed on parental occupation (in models), family history. No ADHD-smoking association.
Ha et al., 2009	6-10 yrs	1778 children from 10 schools in South Korea	Cross-sectional survey (part of prospective cohort)	Geomean (SD): child blood 2.4 (1.96) µg/L	Conners' Parent Rating Scale (ADHD symptoms); (multivariable logistic regression)	Child blood Hg quintile 5 (Hg ≥ 4.5) vs. quintile 1 (Hg < 1.5) µg/L :<br>OR = 0.64, 95% CI 0.28 to 1.48	Models adjusted for multiple confounders including BPb but no information about fish intake.
Surkan et al., 2009	6-10 yrs	355 children from the New England Children's Amalgam Trial (Massachusetts & Maine)	Randomized Clinical Trial (baseline data used for this analysis)	Mean (SD): Child hair at baseline: 0.3 (0.3) µg/g	Multiple psychometric (n=18) measures including IQ, achievement, visual motor & fine motor ability, memory, & executive function.	No significant linear rel'p btw Hg & tests.<br><br>Hg <0.5 → ↑math reasoning; ↑visual-motor skill<br>0.5 ≤ Hg ≤ 1.0 → ↓math reasoning; ↓visual-motor skill<br>Hg > 1.0 (scant data)	Models adjusted for multiple confounders including fish comsumption & BPb. Assessed non-linear dose-response.

Supplemental Material, Table S2 (Cont.) Published Studies of Low-Level Methylmerucy Exposure and Neurocognitive and Behavioral Outcomes (listed by exam age)							
Author	Exam Age	Population/Study Group	Study Design	Mercury Exposure Assessment	Outcomes (analyses)	Findings	Comments
Boucher et al., 2010	10-13 yrs	118 Inuit children in Nunavik, Canada (follow up of Cord Blood Monitoring Program, n=483)	Prospective birth cohort	Median (range): Cord blood 14.2 (1.8-99.3) µg/L Child blood at test 2.8 (0.2-28.1) µg/L	Auditory Event-Related Potentials (ERPs) during oddball task; (hierarchical multivariable linear regression)	↑cord Hg → ↑rxn time & ↓false alarms ↑cord Hg → ↑latency & ↑amplitude (more neg) (target condition)<br><br>No sig rel'p btw child Hg & tests	Models assessed multiple confounders include Pb, DHA, and Se. Hg effects enhances among those breastfed < 3 mos.
Torrente et al., 2005	12-14 yrs	100 children in Terragona, Spain	Cross sectional	Mean (range): Child hair 0.7 (0.1-2.2) µg/g	Standardized testing; (correlation analyses adjusted for age and SES)	Positive correlation (r=0.20, p<0.05) btw child Hg & visuospatial skills	Limited adjustment for potential confounders. Multi-element hair analyses (Cd, Cr, Pb, Mn, Ni, Sn, Hg) assessed one-at-a-time.
Yokoo et al., 2003	17-81 yrs (mean 35)	129 Adults in the Pantanal Region, Brazil	Cross sectional	Median (range); Adult hair 3.7 (0.6-13.6) µg/g	Multiple psychometric tests; (multivariable linear & logistic regression)	↑hair Hg → ↓fine motor speed/dexterity, memory, response inhibition	Limited adjustment for confounders (e.g., fish intake not assessed).
Philibert et al., 2008	18-74 yrs Median: 50 yrs (men) & 47 yrs (women)	243 lake fish eaters in Quebec, Canada	Cross sectional	Median (SD): Adult hair 0.6 (1.4) men; 0.4 (1.0) women µg/g Adult blood 2.3 (5.5) men; 2.1 (3.9) women µg/L	Brief Symptom Inventory (BSI, neuropsychiatric sxs); (multivariate linear regression stratified by sex)	↑Hair Hg → ↑BSI for multiple sxs (e.g., obsessive-compulsive, depression, anxiety) (women only)	Positive results in women only. Essentially no associations with blood Hg. Fish, n-3 PUFAs assessed separately.
Weil et al., 2005	50-70 yrs	Random subset (n=474) from Baltimore Memory Study (n=1140) prospective cohort Baltimore, MD, U.S.A.	Cross-sectional	Median (range): Adult blood 2.1 (0-16) µg/L	12 standardized neurobehavioral tests (20 outcome measures); (multivariable linear regression)	Per IQR ↑Hg:<br><br>↓3% visual memory (Rey Complex Figure delayed recall);<br><br>↑2% manual dexterity (Finger Tapping)	Models considered multiple confounders including fish intake, n-3 PUFAs, BPb, & risk factors for degenerative neurologic disease (e.g., APOE genotype, stroke, diabetes, BMI, homocysteine level, lipids, anithypertension medication, etc.).

Supplemental Material, Table S2 (Cont.) Published Studies of Low-Level Methylmerucy Exposure and Neurocognitive and Behavioral Outcomes (listed by exam age)							
Author	Exam Age	Population/Study Group	Study Design	Mercury Exposure Assessment	Outcomes (analyses)	Findings	Comments
Johansson et al., 2002	≥ 81 yrs, mean 87	Subset (n=106) from Kungsholmen Project prospective cohort	Cross-sectional	Mean (range): Adult blood 3.4 (0.4-16) µg/L	Neurologic exam, Mini-Mental State Examination (MMSE), blood pressure, BMI; (correlation analysis)	Null study (Hg-MMSE correlation = 0.14)	No assessment for confounding. Subsample selected, in part, based on MMSE score. n=8 excluded for 'outlier' Hg values.

Supplemental Material, Table 2 Abbreviations: ADHD (Attention Deficit Hyperactivity Disorder); APOE (apolipoprotein E); BMI (body mass index); BPb (blood lead); btw (between); Cd (cadmium); CI (confidence interval); Cr (chromium); ctrl(s) (control(s)); DHA (docosahexaenoic acid); gen (general); geomean (geometric mean); Hg (mercury); IQ (intelligence quotient); IQR (interquartile range); MeHg (methylmercury); mm (millimeters) ;Mn (manganese); mos (months) ; ms (milliseconds); n-3 PUFA (omega 3 polyunsaturated fatty acid); neg (negative); Ni (nickel); non-sig (non-significant) ; OR (odds ratio); PCBs (polychlorinated biphenyls); pt(s) (point(s)); RBC (red blood cell); re (regarding); rel'p (relationship); resp (respiratory); RR (relative risk) ; rx (medication); rxn (reaction); SD (standard deviation); Se (selenium); SES (socioeconomic status); sig (significant); Sn (tin); sxs (symptoms).

Supplemental Material, Table S3. Published Studies of Low-Level Methylmercury Exposure and Cardiovascular Outcomes (listed by year of publication)

Author	Population/Study Group	Study Design	Sample Size	Exposure Assessment	Exposure Level	Outcomes	Findings	Other contaminants/ nutrients examined
Salonen et al., 1995	Eastern Finnish men (42-60 years)	Prospective cohort	N=1833	Hair and urine Hg	Hair Hg: Mean 1.92 µg/g (Range: 0-15.7 µg/g) Urinary Hg:1.18 µg/24 hr (Range: 0-4.95 µg/24 hr	AMI, death from CHD, CVD	Over 2-fold risk of AMI and mortality from CHD and CVD associated with elevated hair Hg (>2 µg/g)	Intake of fish, iron, plasma fibrinogen, serum selenium and apolipoprotein B, concentrations of HDL_2 cholesterol and ferritin
Salonen et al., 2000	Eastern Finnish men (42-60 years)	Prospective cohort	N=1014	Hair Hg	Mean: 1.8 µg/g (Range: 0-23.3 µg/g)	Carotid atherosclerosis (determined by intima-media thickness, IMT)	Hg associated with accelerated progression of carotid atherosclerosis.	Intake of cholesterol, selenium and unspecified fatty acids, fibers, and vitamins
Rissanen et al., 2000	Eastern Finnish men (42-60 years)	Prospective cohort	N=1871	Hair Hg	Mean: 1. 91 µg/g (Range: 0-15.67 µg/g)	Fatal or nonfatal acute coronary events	Fish oil-derived fatty acids reduce risk of acute coronary events; high Hg in fish could attenuate this effect.	DPA+DHA, EPA, serum ferritin, serum LDL cholesterol, serum insulin
Guallar et al., 2002	Men from eight European countries and Israel (70 years or younger) Avg. age: Cases: 54.7±8.9yrs; Cntrls:53.2±9.3 yrs	Case-control study	Cases N=684; Controls N=724	Toenail Hg	Overall means: Cases = 0.27 µg/g (Range 0.14-0.68 µg/g); Controls = 0.25 µg/g (Range 0.14-0.57 µg/g)	First diagnosis of MI	Toenail Hg associated with risk of MI; DHA inversely associated with risk.	DHA, serum cholesterol

Supplemental Material, Table S3 (Cont.) Published Studies of Low-Level Methylmercury Exposure and Cardiovascular Outcomes (listed by year of publication)

Author	Population/Study Group	Study Design	Sample Size	Exposure Assessment	Exposure Level	Outcomes	Findings	Other contaminants/ nutrients examined
Yoshizawa et al., 2002	US male health professionals (40-75 years)	Nested case-control with five years of follow-up	470 cases (from 33,737 members); 464 controls (matched by age and smoking status)	Toenail Hg	Mean Hg, Cases = 0.74 µg/g; Controls = 0.72 µg/g	CHD (coronary-artery surgery, nonfatal MI, fatal CHD)	No association between Hg and risk of CHD; after excluding dentists, RR for highest exposure (0.84 µg/g) versus lowest (0.13 µg/g) = 1.27, not statistically significant.	Levels of DHA, EPA, selenium, and cadmium
Virtanen et al. 2005	Eastern Finnish men (42-60 years)	Prospective cohort	N=1871	Hair Hg	Mean=1.9 µg/g (Range: 0-15.7 µg/g)	Acute coronary events and cardiovascular and all-cause mortality	Increased Hg exposure associated with increased risk of acute coronary events and CVD mortality; Hg seemed to attenuate the protective effects of fish on cardiovascular health.	DHA+DPA, HDL and LDL cholesterol, serum selenium, dietary intakes of saturated fatty acids, fiber, and vitamins C and E
Vupputuri et al., 2005	NHANES 1999-2000 Women (16-49 years)	Cross-sectional survey of the US population	N=1240	Blood Hg	Mean=1.8 µg/L (Range: 0.1-21.4 µg/L Fish consumers, Mean = 2.3 µg/L; Non-fish consumers, Mean=0.8 µg/L)	Systolic and diastolic blood pressure	Hg not significantly associated with SBP or DBP in entire cohort; however, significant positive association between SBP and Hg among non-fish consumers (similar pattern for DBP, non-significant).	Intake of sodium, potassium, and fish

Supplemental Material, Table S3 (Cont.) Published Studies of Low-Level Methylmercury Exposure and Cardiovascular Outcomes (listed by year of publication)

Author	Population/Study Group	Study Design	Sample Size	Exposure Assessment	Exposure Level	Outcomes	Findings	Other contaminants/ nutrients examined
Valera et al., 2009	Nunavik Inuit (18-71 years)	Prospective cohort	N=732	Blood Hg	Mean: 10.2 µg/L	BP, and pulse pressure	Hg associated with increasing BP and pulse pressure.	DHA, EPA, selenium, and lead levels, LDL and HDL cholesterol, triglycerides
Mozaffarian et al., 2011	HPFS (male US health professionals), 40-75 years and NHS (female US registered nurses), 30-55 years	Nested case control study from both prospective cohort studies	Cases: N=3427 Controls: N=3427	Toenail Hg	Mean (SD): Men: Cases= 0.51 µg/g (2.13); Controls=0.44 µg/g (0.47) Women: Cases=0.29 µg/g (0.49); Controls=0.33 µg/g (0.63)	Incident CVD (i.e. nonfatal MI, fatal CHD or stroke)	No adverse effects of Hg exposure on CHD, stroke, or total CVD.	Selenium, consumption of fish, DHA, EPA
Wennberg et al., 2011	Northern Sweden cohort	Nested case control study	Cases: N=431 Controls: N=499	Ery-Hg	Median (range) of Ery-Hg: 3.54 (0.01-87) µg/g or 0.52 (0.0015-12.7) µg/g hair-Hg	Myocardial infarction cases including sudden cardiac death	No adverse effect of mercury on the risk of myocardial infarction	Selenium, EPA, DHA

Supplemental Material, Table 3 Abbreviations: AMI (acute myocardial infarction); CHD (coronary heart disease); CVD (cardiovascular disease); DBP (diastolic blood pressure); DHA (docosahexaenoic acid); DPA (docosapentaenoic acid); EPA (eicosapentaenoic acid); Ery-Hg (erythrocyte mercury); Hg (mercury); HPFS (Health Professionals Follow-up Study); IMT (intema media thickness); MI (myocardial infarction); NHANES (National Health and Nutrition Examination Survey); NHS (Nurse's Health Study); RR (relative risk); SBP (systolic blood pressure)

**Supplemental Material, Table S4.** Published Studies of Low-Level Methylmercury Exposure and Immunologic Outcomes (listed by year of publication)							
Author	Population/ Study Group	Study Design	Sample Size	Exposure Assessment	Exposure Level	Findings	Effect Modifiers/Adjustment for Confounders
Belles-Isles et al., 2002	Newborns in Canadian subsistence fishing population & coastal town residents (reference group)	Cross sectional (births 1995-97)	Mother-infant pairs: n=48 (subsistence fishers) & n=60 (coastal town residents)	Cord blood	Cord blood Geomean (95% CI): 1.8 (1.4-2.3) µg/L (subsistence fishers); 0.9 (0.8-1.0) µg/L (coastal town residents)	Cord blood Hg inversely correlated with proportion of naive helper T cells and plasma IgM levels in cord blood. No relationship with multiple other measures of T, B, and NK (natural killer) cell proportions and function	No adjustment for confounders despite substantial differences between subsistence fishers and coastal residents, including organochlorine exposures
Bilrha et al., 2003	Newborns in Canadian subsistence fishing population &coastal town residents (reference group)	Cross sectional (births 1997-98)	Mother-infant pairs: n=47 (subsistence fishers) n=65 (coastal town residents)	Cord blood	Cord blood Hg Geomemean (95% CI): 1.8 (1.5-2.2) µg/L (subsistence fishers) 1.1 (0.9-1.2) µg/L (coastal town residents)	No correlation between cord blood Hg & cord blood lymphocyte activation markers or cytokine secretion.	No adjustment for confounders despite substantial differences between subsistence fishers and coastal residents, including organochlorine exposures
Miyake et al., 2011	Osaka Maternal and Child Health Study	Prospective prebirth cohort	582 mother/child pairs	Maternal hair andchild hair at 29-39 months	Maternal hair Hg Median 1.52 µg/g Range 0.26 – 6.05 µg/g<br>Child hair Hg Median 1.38 µg/g Range 0.13 – 9.51µg/g	No association between hair mercury and risk of wheeze or eczema in children. Suggestive but non-significant eczema risk with OR = 1.26, 95% CI 0.67-2.36 for highest vs. lowest quartile of child hair Hg	Models adjusted for potential confounders including maternal pregnancy and child fish intake. Despite adjustment, authors consider there to be potential residual confounding by fish intake.

**Supplemental Material, Table S4 (Cont.)** Published Studies of Low-Level Methylmercury Exposure and Immunologic Outcomes (listed by year of publication)							
Author	Population/ Study Group	Study Design	Sample Size	Exposure Assessment	Exposure Level	Findings	Effect Modifiers/Adjustment for Confounders
Nyland et al., 2011	Mother-infant pairs in Brazilian Amazon	Population-based Survey	61 mother-infant pairs	Cord blood and maternal blood at delivery	Cord blood Hg: 9.63 µg/L Range: 0.08-77.80 µg/L<br><br>Maternal blood Hg: 6.90 µg/L Range: 0.08-55.48 µg/L	Total IgG level in cord blood significantly positively associated with cord blood and maternal Hg levels.<br>No associations seen with serum ANA titers or cytokine levels.	Adjusted for maternal age, education level, and residence (no fish consumption information but education correlated with fish intake).
Park & Kim, 2011	General adult population (≥20 years) in Republic of Korea	Population based cross sectional survey	1990 Korean adults [11% with lifetime s AD; 9% with AD within 1yr of study]	Adult blood	Geomean, no AD: 3.45 µg/L (95% CI 3.17-3.76)<br>Geomean, lifetime AD: 4.66 µg/L (95% CI 3.81-5.70)<br>Geomean, 1 yr AD: 4.91 µg/L (95% CI 3.97-6.08)	Hg significantly associated with increased lifetime and 1 yr AD:<br>OR (95% CI) lifetime AD, upper vs. lower tertile Hg: 1.50 (1.02-2.21)<br>OR 1 yr AD, upper vs. lower tertile Hg: 1.82 (1.17-2.83)	Adjusted for multiple potential confounders including fish &shellfish intake.
Supplemental Material, Table 4 Abbreviations: AD (atopic dermatitis); ANA (antinuclear autoantibody); BMI (body mass index); BPb (blood lead); CI (confidence interval); DDE (dichlorodiphenyldichloroethylene); geomean (geometric mean); Hg (mercury); ;med (medium); mos (months); OR (odds ratio); n-3 PUFAs (omega-3 polyunsaturated fatty acids); PCBs (polychlorinated biphenyls); SES (socioeconomic status)							

REFERENCS

Barbone F, Valent F, Pisa F, Daris F, Fajon V, Ing D, et al. 2004. Prenatal low-level methlmercury exposure and child development in an Italian costal area. Seychelles Medical and Dental Journal (SMDJ) 7(1): 149-154.

Belles-Isles M, Ayotte P, Dewailly E, Weber J-P, Roy R. 2002. Cord blood lymphocyte functions in newborns from a remote maritime population exposed to organochlorines and methylmercury. J Toxicol Environ Health A 65:165-182.

Bilrha H, Roy R, Moreau B, Belles-Isles M, Dewailly E, Ayotte P. 2003. In vitro activation of cord blood mononuclear cells and cytokine production in a remote coastal population exposed to organochlorines and methyl mercury. Environ Health Perspect 111(16): 1952-1957.

Boucher O, Bastien CH, Saint-Amour D, Dewailly E, Ayotte P, Jacobson JL, et al. 2010. Prenatal exposure to methylmercury and PCBs affects distinct stages of information processing: an event-related potential study with Inuit children. Neurotoxicology 31(4): 373-384.

Cace IB, Milardovic A, Prpic I, Krajina R, Petrovic O, Vukelic P, et al. 2011. Relationship between the prenatal exposure to low-level of mercury and the size of a newborn's cerebellum. Med Hypotheses 76(4): 514-516.

Cao Y, Chen A, Jones RL, Radcliffe J, Caldwell KL, Dietrich KN, et al. 2010. Does background postnatal methyl mercury exposure in toddlers affect cognition and behavior? Neurotoxicology 31(1): 1-9.

Cheuk DK, Wong V. 2006. Attention-deficit hyperactivity disorder and blood mercury level: a case-control study in Chinese children. Neuropediatrics 37(4): 234-240.

Daniels JL, Longnecker MP, Rowland AS, Golding J. 2004. Fish intake during pregnancy and early cognitive development of offspring. Epidemiology 15(4): 394-402.

Daniels JL, Rowland AS, Longnecker MP, Crawford P, Golding J. 2007. Maternal dental history, child's birth outcome and early cognitive development. Paediatr Perinat Epidemiol 21(5): 448-457.

Despres C, Beuter A, Richer F, Poitras K, Veilleux A, Ayotte P, et al. 2005. Neuromotor functions in Inuit preschool children exposed to Pb, PCBs, and Hg. Neurotoxicol Teratol 27(2): 245-257.

Drouillet-Pinard P, Huel G, Slama R, Forhan A, Sahuquillo J, Goua V, et al. 2010. Prenatal mercury contamination: relationship with maternal seafood consumption during pregnancy and fetal growth in the 'EDEN mother-child' cohort. Br J Nutr 104(8): 1096-1100.

Freire C, Ramos R, Lopez-Espinosa MJ, Diez S, Vioque J, Ballester F, et al. 2010. Hair mercury levels, fish consumption, and cognitive development in preschool children from Granada, Spain. Environ Res 110(1): 96-104.

Gao Y, Yan CH, Tian Y, Wang Y, Xie HF, Zhou X, et al. 2007. Prenatal exposure to mercury and neurobehavioral development of neonates in Zhoushan City, China. Environ Res 105(3): 390-399.

Guallar E, Sanz-Gallardo MI, van't Veer P, Bode P, Aro A, Gomez-Aracena J, et al. 2002. Mercury, fish oils, and the risk of myocardial infarction. N Engl J Med 347(22): 1747-1754.

Gundacker C, Frohlich S, Graf-Rohrmeister K, Eibenberger B, Jessenig V, Gicic D, et al. 2010. Perinatal lead and mercury exposure in Austria. Sci Total Environ 408(23): 5744-5749.

Ha M, Kwon HJ, Lim MH, Jee YK, Hong YC, Leem JH, et al. 2009. Low blood levels of lead and mercury and symptoms of attention deficit hyperactivity in children: a report of the children's health and environment research (CHEER). Neurotoxicology 30(1): 31-36.

Jedrychowski W, Jankowski J, Flak E, Skarupa A, Mroz E, Sochacka-Tatara E, et al. 2006. Effects of prenatal exposure to mercury on cognitive and psychomotor function in one-year-old infants: epidemiologic cohort study in Poland. Ann Epidemiol 16(6): 439-447.

Jedrychowski W, Perera F, Rauh V, Flak E, Mroz E, Pac A, et al. 2007. Fish intake during pregnancy and mercury level in cord and maternal blood at delivery: an environmental study in Poland. Int J Occup Med Environ Health 20(1): 31-37.

Johansson N, Basun H, Winblad B, Nordberg M. 2002. Relationship between mercury concentration in blood, cognitive performance, and blood pressure, in an elderly urban population. Biometals 15(2): 189-195.

Kim BM, Lee BE, Hong YC, Park H, Ha M, Kim YJ, Kim Y, Chang N, Kim BN, Oh SY, Yoo M, Ha EH. 2011. Mercury levels in maternal and cord blood and attained weight through the 24 months of life. Sci Total Environ 410: 26-33.

Lederman SA, Jones RL, Caldwell KL, Rauh V, Sheets SE, Tang D, et al. 2008. Relation between cord blood mercury levels and early child development in a World Trade Center cohort. Environ Health Perspect 116(8): 1085-1091.

Lee BE, Hong YC, Park H, Ha M, Koo BS, Chang N, et al. 2010. Interaction between GSTM1/GSTT1 polymorphism and blood mercury on birth weight. Environ Health Perspect 118(3): 437-443.

Lucas M, Dewailly E, Muckle G, Ayotte P, Bruneau S, Gingras S, et al. 2004. Gestational age and birth weight in relation to n-3 fatty acids among Inuit (Canada). Lipids 39(7): 617-626.

Miyake Y, Tanaka K, Yasutake A, Sasaki S, Hirota Y. 2011. Lack of association of mercury with risk of wheeze and eczema in Japanese children: The Osaka Maternal and Child Health Study. Environ Res 111(2011): 1180-1184.

Mozaffarian D, Shi P, Morris JS, Spiegelman D, Grandjean P, Siscovick DS, et al. 2011. Mercury exposure and risk of cardiovascular disease in two U.S. cohorts. N Engl J Med 364(12): 1116-1125.

Nyland JF, Wang SB, Shirley DL, Santos EO, Ventura AM, Souza JM, et al. 2011. Fetal and maternal immune responses to methylmercury exposure: a cross-sectional study. Environ Res. 111(2011): 584-589.

Oken E, Radesky JS, Wright RO, Bellinger DC, Amarasiriwardena CJ, Kleinman KP, et al. 2008. Maternal fish intake during pregnancy, blood mercury levels, and child cognition at age 3 years in a US cohort. Am J Epidemiol 167(10): 1171-1181.

Oken E, Wright RO, Kleinman KP, Bellinger D, Amarasiriwardena CJ, Hu H, et al. 2005. Maternal fish consumption, hair mercury, and infant cognition in a U.S. Cohort. Environ Health Perspect 113(10): 1376-1380.

Park H and Kim K. 2011. Association of blood mercury concentrations with atopic dermatitis in adults: a population-based study in Korea. Environ Res 111(2011): 573-578.

Philibert A, Bouchard M, Mergler D. 2008. Neuropsychiatric symptoms, omega-3, and mercury exposure in freshwater fish-eaters. Arch Environ Occup Health 63(3): 143-153.

Plusquellec P, Muckle G, Dewailly E, Ayotte P, Begin G, Desrosiers C, et al. 2010. The relation of environmental contaminants exposure to behavioral indicators in Inuit preschoolers in Arctic Quebec. Neurotoxicology 31(1): 17-25.

Ramon R, Ballester F, Aguinagalde X, Amurrio A, Vioque J, Lacasana M, et al. 2009. Fish consumption during pregnancy, prenatal mercury exposure, and anthropometric measures at birth in a prospective mother-infant cohort study in Spain. Am J Clin Nutr 90(4): 1047-1055.

Rissanen T, Voutilainen S, Nyyssonen K, Lakka TA, Salonen JT. 2000. Fish oil-derived fatty acids, docosahexaenoic acid and docosapentaenoic acid, and the risk of acute coronary events: the Kuopio ischaemic heart disease risk factor study. Circulation 102(22): 2677-2679.

Saint-Amour D, Roy MS, Bastien C, Ayotte P, Dewailly E, Despres C, et al. 2006. Alterations of visual evoked potentials in preschool Inuit children exposed to methylmercury and polychlorinated biphenyls from a marine diet. Neurotoxicology 27(4): 567-578.

Salonen JT, Seppanen K, Lakka TA, Salonen R, Kaplan GA. 2000. Mercury accumulation and accelerated progression of carotid atherosclerosis: a population-based prospective 4-year follow-up study in men in eastern Finland. Atherosclerosis 148(2): 265-273.

Salonen JT, Seppanen K, Nyyssonen K, Korpela H, Kauhanen J, Kantola M, et al. 1995. Intake of mercury from fish, lipid peroxidation, and the risk of myocardial infarction and coronary, cardiovascular, and any death in eastern Finnish men. Circulation 91(3): 645-655.

Sikorski R, Paszkowski T, Szprengier-Juszkiewicz T. 1986. Mercury in neonatal scalp hair. Sci Total Environ 57: 105-110.

Stewart PW, Reihman J, Lonky EI, Darvill TJ, Pagano J. 2003. Cognitive development in preschool children prenatally exposed to PCBs and MeHg. Neurotoxicol Teratol 25(1): 11-22.

Surkan PJ, Wypij D, Trachtenberg F, Daniel DB, Barregard L, McKinlay S, et al. 2009. Neuropsychological function in school-age children with low mercury exposures. Environ Res 109(6): 728-733.

Suzuki K, Nakai K, Sugawara T, Nakamura T, Ohba T, Shimada M, et al. 2010. Neurobehavioral effects of prenatal exposure to methylmercury and PCBs, and seafood intake: neonatal behavioral assessment scale results of Tohoku study of child development. Environ Res 110(7): 699-704.

Torrente M, Colomina MT, Domingo JL. 2005. Metal concentrations in hair and cognitive assessment in an adolescent population. Biol Trace Elem Res 104(3): 215-221.

Valera B, Dewailly E, Poirier P. 2009. Environmental mercury exposure and blood pressure among Nunavik Inuit adults. Hypertension 54(5): 981-986.

Virtanen JK, Voutilainen S, Rissanen TH, Mursu J, Tuomainen TP, Korhonen MJ, et al. 2005. Mercury, fish oils, and risk of acute coronary events and cardiovascular disease, coronary heart disease, and all-cause mortality in men in eastern Finland. Arterioscler Thromb Vasc Biol 25(1): 228-233.

Vupputuri S, Longnecker MP, Daniels JL, Guo X, Sandler DP. 2005. Blood mercury level and blood pressure among US women: results from the National Health and Nutrition Examination Survey 1999-2000. Environ Res 97(2): 195-200.

Weil M, Bressler J, Parsons P, Bolla K, Glass T, Schwartz B. 2005. Blood mercury levels and neurobehavioral function. Jama 293(15): 1875-1882.

Wennberg M, Bergdahl IA, Hallmans G, Norberg M, Lundh T, Skerfving S, Strömberg U, Vessby B, Jansson JH. 2011. Fish consumption and myocardial infarction: a second prospective biomarker study from northern Sweden. Am J Clin Nutr. 93(1):27-36.

Xue F, Holzman C, Rahbar MH, Trosko K, Fischer L. 2007. Maternal fish consumption, mercury levels, and risk of preterm delivery. Environ Health Perspect 115(1): 42-47.

Yokoo EM, Valente JG, Grattan L, Schmidt SL, Platt I, Silbergeld EK. 2003. Low level methylmercury exposure affects neuropsychological function in adults. Environ Health 2(1): 8.

Yoshizawa K, Rimm EB, Morris JS, Spate VL, Hsieh CC, Spiegelman D, et al. 2002. Mercury and the risk of coronary heart disease in men. N Engl J Med 347(22): 1755-1760.
